# Reinforcement of Nanocomposite Hydrogel with Dialdehyde Cellulose Nanofibrils via Physical and Double Network Crosslinking Synergies

**DOI:** 10.3390/polym15071765

**Published:** 2023-04-01

**Authors:** Liang Li, Jixiang Guo, Chuanhong Kang, Hanxuan Song

**Affiliations:** Unconventional Oil and Gas Institute, China University of Petroleum, Beijing 102249, China; 2018310102@student.cup.edu.cn (L.L.); k15616309001@163.com (C.K.); 2019215169@student.cup.edu.cn (H.S.)

**Keywords:** cellulose nanofibril, high-strength, double network crosslinking, nanocomposite hydrogel, reinforcement, polymers

## Abstract

Preparation of tough and high-strength hydrogels for water plugging in oil fields with an easy-scalable method is still considered to be a challenge. In this study, dialdehyde cellulose nanofibril (DA-CNF) prepared by sodium periodate oxidation, polyamine, 2-acrylamido-2-methylpropane sulfonic acid (AMPS) with sulfonate groups and Acrylamide (AM) as raw materials, CNF reinforced nanocomposite hydrogels were prepared in one step by in-situ polymerization. The tensile strength, and texture stability of the obtained nanocomposite hydrogel were determined. The results showed that the tensile strength and toughness of the obtained nanocomposite hydrogel increased four times compared with control sample due to physical and chemical double crosslinking synergies. Moreover, the texture intensity of DA-CNFs reinforced hydrogel still maintains high stability and strength performance under high salinity conditions. Therefore, DA-CNF reinforced hydrogel has potential application value in both normal and high-salinity environments in oil recovery.

## 1. Introduction

Hydrogel is a kind of gel with a three-dimensional network structure, composed of hydrophilic polymers, which has been widely used in various areas, such as sanitary products, agriculture, ocean industry, biomedical, and petroleum exploitation due to its swell ability and special mechanical properties (wet-strength and flexibility) [1,2,3,4,5,6,7,8]. However, the traditional hydrogel has some shortcomings, such as insufficient mechanical strength, inapplicability to high temperature and high salt conditions, which severely limits its application and development. The challenge of further utilization of hydrogels in plugging agent, however, is the relative low mechanical strength. To improve the hydrogel strength, a variety of strategies, including preparation of double network hydrogel, ionic crosslinked hydrogel, and nanocomposite hydrogel have been investigated [9,10]. Nanocomposite hydrogel refers to the method of preparing hydrogel by blending and polymerizing natural polymer chains, nano reinforcement materials, and polymer monomers, and using the excellent properties of nano materials and particles to enhance the performance of hydrogel. For instance, nanocomposite hydrogels are envisioned to have great potential for advanced applications due to their simple production method, excellent mechanical performance, and multi-sensing capacity [11,12,13].

Cellulose fibers are deconstructed and peeled off by chemical and physical methods to reach nanometer level in a certain dimension. This kind of cellulose is called nanometer cellulose. According to different preparation conditions, size, and morphology, different types of nanocellulose can be formed, which can be divided into cellulose nanocrystals, cellulose nanofibers, and bacterial nanocellulose. Nanocellulose has the characteristics of high modulus, high aspect ratio, high strength, and good biocompatibility, making it an excellent nano-reinforced material. Nanocellulose, produced from renewable biomass, is a sustainable and environmentally friendly raw material with several unique properties such as biocompatibility, non-toxicity, biodegradability, and reproducibility [14,15]. As a nano-scale material, nanocellulose has a large specific surface area that allows the good interaction with polymers, nanoparticles, and other small molecules; this makes nanocellulose a potential reinforcing agent for the production of high-strength nanocomposite hydrogels [16]. Moreover, due to the high content of hydroxyl group in the cellulose structure, the chemical property of nanocellulose can also be easily tuned through chemically functionalization. Therefore, the modification of nanocellulose to improve the physical strength of hydrogels is a promising strategy for expanding both the application of nanocellulose and hydrogels.

Compared to other kinds of nanocellulose such as cellulose nanocrystals and bacterial nanocellulose, cellulose nanofibrils (CNFs) with a length of 10–20 μm and width of 20–30 nm are recognized as a good starting material to prepare high-strength hydrogels related to its high chemical flexibility to interweave and entangle in the polymer chain network [17]. The reinforcement of hydrogels with CNFs was mainly through physical cross-linking such as hydrogen bonding and entanglement [16]. In addition, the addition of metal ions or modifying CNF might improve the reinforcement effect of CNFs by forming some chemical cross-links between CNFs and polymer chains, thereby yielding a more stable semi-interpenetrating network [18]. For example, Zheng et al. report the aldehyde modified CNFs could improve the hydrogel compression strength by 5800% [19]. However, the work on the production of high-strength hydrogels reinforced by CNFs that directly construct ion crosslinking between CNF and polymer chains has not been reported.

In this work, sodium periodate oxide CNFs crosslinked with polyamine to reinforce the poly(acrylamide-co-2-acrylamido-2-methylpropane sulfonic acid) [P(AM-co-AMPS)] hydrogel through an in-situ polymerization approach was investigated. The work was built on the proposed mechanism that forming double networks by polyamine and DA-CNFs and AM and AMPS through different chemical crosslinks to improve the strength properties of the nanocomposite hydrogel. The effect of DA-CNFs on the internal network structure of nanocomposite hydrogels was studied by scanning electron microscope (SEM). Moreover, the proposed mechanism was also proved by the tensile strength test. In addition, the results on the improved mechanical strength of the hydrogel also demonstrated that the synergistic effect of chemical and physical crosslinking between DA-CNFs and polymer matrix occurred. The swelling behavior and texture intensity of hydrogels in high salinity solution was also studied.

## 2. Materials and Methods

### 2.1. Materials

The nanofibrils (CNF) suspension (solid consistency of 2.0 wt%), produced from softwood bleached kraft pulp via the mechanical refining process, were provided by Tianjin Woodelf Biotechnology Co., Ltd. (Tianjin, China). 1-Hydroxycyclohexyl phenyl keton (V50), Acrylamide (AM), 2-Acrylanmido-2-methylpropanesulfonic acid (AMPS), N,N′-Methylenebis(2-propenamide) (MBA) and was purchased from Sinopharm (Beijing, China), polyamine (D230) was purchased from BASF SE. All chemicals used in this study were reagent grade and utilized without further purification.

### 2.2. Preparation of Dialdehyde Cellulose Nanofibrils (DA-CNFs)

The dialdehyde oxidation of the CNFs was performed in a 1000-mL beaker, in which 20 g (dry weight) of CNF, 10 g sodium periodate, and 950 mL of deionized water were mixed and adjust pH to 5.0, then immersed in a water bath preheated to 50 °C and the mixture was then stirred for 6 h. After the completion of the oxidation, the solid substance was washed with water to remove the traces of sodium periodate and acid. The dialdehyde group of DA-CNF was determined by the hydroxylamine hydrochloride method [20]. In brief, 1.0 g of DA-CNF was mixed with 200 mL of 0.2 M hydroxylamine hydrochloride solution at pH 3 and dispersed by high speed disperser at 20,000 rpm for 3 min. The DA-CNF solution was titrated to pH 3 using a 0.01 M KOH solution. The control sample was performed similarly with non-oxidized CNF and the aldehyde content determined from:

(1) $$\mathrm{Dialdehyde}\;\mathrm{group}\;\mathrm{content}=\frac{\left(V_{1}-V_{2}\right)\times C_{KOH}}{m}$$

where *V*_1_ is volume of sample consumed *C_KOH_* and *V*_2_ is volume of the control sample consumed *C_KOH_* is the concentration of KOH solution and *m* the weight of the dry DA-CNF or control CNF.

### 2.3. Preparation of Nanocomposite Hydrogel

During the preparation of DA-CNF-based nanocomposite hydrogel, the mass ratios of AM, AMPS, polyamine of 65:25:10, and DA-CNF addition was 1.0% to 5%. For each hydrogel preparation run, AM, AMPS, polyamine and DA-CNF were mixed and diluted to a concentration of 30% (*w*/*v*) with deionized water. Then, DA-CNFs were dispersed into the solution using a high speed disperser at 20,000 rpm for 3 min. Then, MBA, V50 were added into the mixture at loadings of 0.10, and 0.1 g per 100 g (dry weight) polymer substrate (AM and AMPS), respectively. Then, the polymerization process was initiated using an ultraviolet light at 250 W for 20 min to form poly(acrylamide-co-2-acrylamido-2-methylpropane sulfonic acid)/polyamine and DA-CNF [P(AM-co-AMPS)/PA-DA-CNF] hydrogel. The without DA-CNF hydrogel was prepared in the same way and utilized as the control. The formed P(AM-co-AMPS)/PA-DA-CNF hydrogel was put in the oven at 105 °C until to oven dry, after that the samples were blended and sifted into particle size: <60–120 mesh.

### 2.4. Characterization of Hydrogel

Before the analysis, the hydrogel was extracted using a Soxhlet apparatus by DI water. A total of 2.0 g hydrogel was placed into a Whatman cellulose thimble and Soxhlet extraction system, extracted with deionized water for 10 h to remove the non-crosslinked components, such as AM, AMPS, and polyamine. 

Fourier Transform Infrared Spectroscopy (FTIR) analysis of the hydrogel samples was conducted with a FTIR-650 spectrometer (Gangdong Co., Ltd., Tianjin, China) in the wave number range of 4000–400 cm^−1^.

The X-ray Photoelectron Spectroscopy (XPS) characterizations were done with a ESCALAB 250Xi XPS microprobe (Thermo Fisher Scientific, Waltham, MA, USA) with radiation (0–5000 eV). Spectrum NT and the CAH signal were used to deconvolute the peaks. 

Atomic force microscopy (AFM) was recorded with an Atomic Force Microscope (MultiMode 8, Bruker, Billerica, MD, USA) in tapping mode. For the measurement, a drop of the CNF suspension (consistency of 0.01%) was placed onto a clean mica sheet and air-dried.

### 2.5. Analytical Methods

The mechanical strength of hydrogel was determined using a tensile tester (INSTRON 3366, Canton, MA, USA) at room temperature. For the determination of tensile stress–strain, samples were cut into a dumbbell shape with size of 3 × 4 × 25 mm for thickness × width × gauge length to measure tensile properties. The extension rate was fixed at 130 mm/min for uniaxial tensile tests. The tensile strength and stretch were determined by the failure point. The Elastic modulus numerical value was obtained as the slope in a linear part of the initial portion of the stress–strain curves (5–15% strain).

The compression tests were conducted with the cylindrical hydrogel samples (40 mm in diameter and 15 mm in height) at a compression rate of 8 mm/min until the compression deformation reached 60%, the modulus was obtained from the slope of stress–strain curve from 20–30% of the strain ratio. The cyclic compression for eight times was determined with the same deformation. The hysteresis energy is calculated by the area enclosed by the loading curve and the unloading curve using integration. All experiments were performed at least in triplicate.

The texture analyzer (TA-XT plus, Stable Micro Systems, Godalming, Surrey, UK) was used to test the strength of composite hydrogel particles under different compression ratios. The swollen hydrogel was placed on the tray of the texture analyzer, with a test area of 30 mm × 30 mm. During the process, compression speed is 5 mm/min, force is recorded to measure the strength of composite hydrogel particles. The recoverability of composite hydrogel particles is defined as the ratio of the integral area under the stress curve in the rising process to the integral area under the stress curve in the falling process.

Before testing the swelling property, the particles were placed in an oven at 60 °C for 48 h to evaporate the water. The 10% (*w*/*w*) salt solution was prepared by dissolving the mixed the salts of m (NaCl), m (CaCl_2_), and m (MgCl_2_) at a ratio of 6:1:1 into distilled water. The swollen gels were removed from the salt solution at different time intervals (1 to 16 days) and dried superficially with a paper filter. The weight of each dry gel was measured and placed back into the same bath until a constant weight was reached for each sample. Three replicates were performed for each hydrogel and the average value was reported. The percent of swelling of each hydrogel was calculated in the form of water uptake quantities *Q* (g/g) following Equation (1):

(2) $$Q=\frac{m_{t}-m_{0}}{m_{0}}$$

where *m_t_* is the weight of swollen gel at time *t*, and *m*_0_ is the initial weight of the dry gel.

## 3. Results and Discussion

### 3.1. Synthesis of Hydrogels

**Figure 1:**
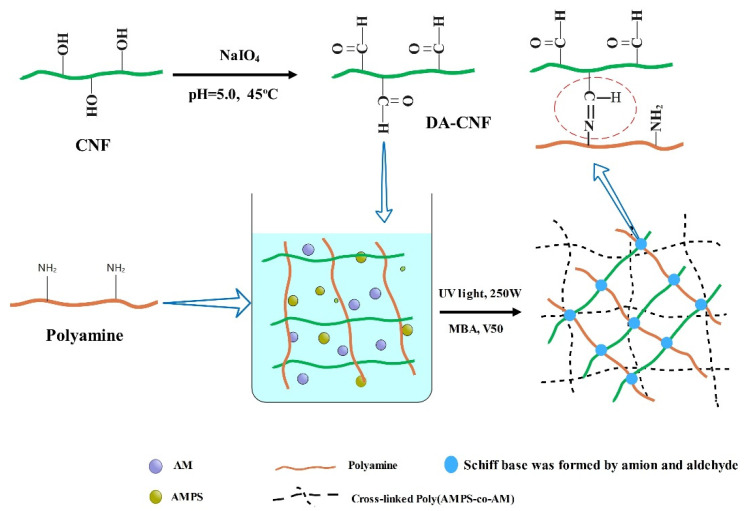
Schematic illustration of dialdehyde cellulose nanofibrils preparation and the synthesis of P(AM-co-AMPS)/PA/DA-CNF hydrogel.

Figure 1 shows a schematic diagram for the preparation of the P(AM-co-AMPS)/PA/DA-CNF hydrogel. As shown, to construct the nanocomposite hydrogel system with the interaction with double networks, polyamine and DA-CNFs were used as interaction objects while AM and AMPS were applied to provide strength of the hydrogel. CNFs were firstly dialdehyded through sodium periodate oxidation by broke C2 and C3 groups on CNFs [21,22]. Then, AMPS, AM, DA-CNF, and polyamine were mixed and initiated with UV light to form nanocomposite hydrogel that contains double network through in situ polymerization. In this hydrogel, AM and AMPS were polymerized to form a network polymer, which was functionalized as the basic framework of the hydrogel. In addition, the aldehyde group of the DA-CNFs, reacted with the amino group in the polyamine chain, could further strengthen the network [23]. The double network in this semi-interpenetrating nanocomposite hydrogel system is expected to substantially improve the mechanical properties due to the synergistic effect between the two crosslinking methods. The hydrogels of P(AM-co-AMPS) without DA-CNFs and P(AM-co-AMPS)/PA/DA-CNF without double network crosslinking were also prepared and investigated as the control.

**Figure 2:**
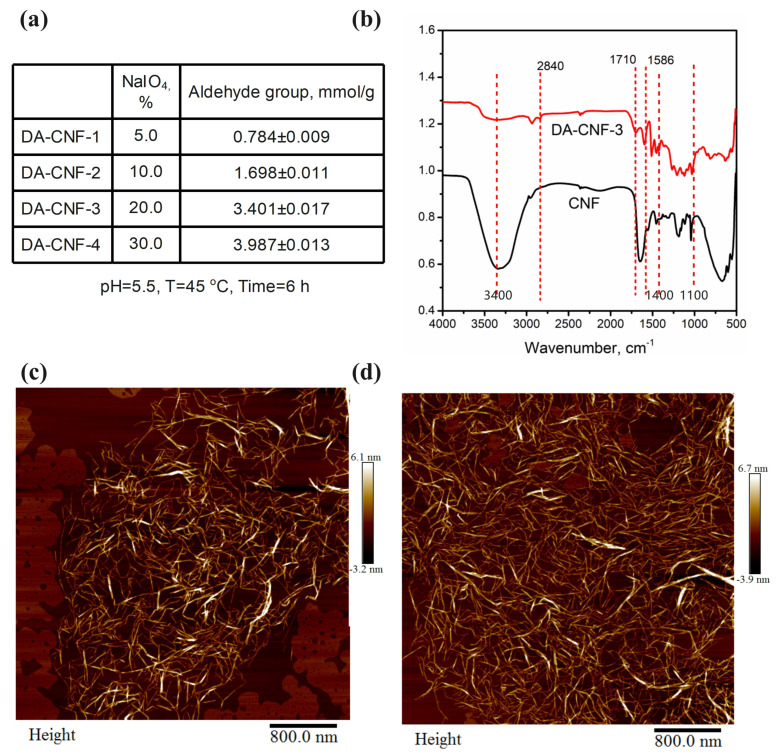
Characteristics of DA-CNF. (**a**) Oxidated CNF by sodium periodate. (**b**) FTIR spectra of CNF and DA-CNF. (**c**) AFM height images of CNF. (**d**) AFM height images of DA-CNF.

In addition, the content of aldehyde group on DA-CNFs was determined, with the increasing of the sodium periodate addition, the aldehyde group content was increased. The amount of sodium periodate oxidation was determined by measuring the aldehyde group content. By adjusting the loading of sodium periodate, four different amounts of DA-CNF were obtained: 0.784 ± 0.009 mmol/g, 1.5698 ± 0.011 mmol/g, 3.401 ± 0.017 mmol/g, and 3.987 ± 0.013 mmol/g. Generally, aldehyde content increased is a benefit to cross-linking polymerization, in the subsequent study, DA-CNF with 3.987 ± 0.013 mmol/g was chosen as the additive in polymerization. Figure 2b shows the FTIR spectra of the CNFs and DA-CNFs. Both of the two spectra displayed diagnostic peaks at 3300, 2900, 1586, and 1100 cm^−1^, which were attributed to the vibration frequencies of OH stretching, CH_2_ stretching, OH bending vibrations, and C–O stretching, respectively [20,24], while the peaks at 1400 and 1021 cm^−1^ were attributed to CH_,_ bending and C–O–C stretching vibrations. An absorption peak at 1710 cm^−1^, was attributed to the aldehyde group of the DA-CNFs [20]. These results together convinced the successful oxidated CNF.

Figure 2c and d shows the morphology of the CNF and DA-CNF samples. As shown, the size and dispersion of the CNFs and DA-CNFs were essentially identical, indicating that the oxidation could preserve the fiber morphology. Moreover, the aspect ratio of DA-CNFs was mainly in the range of 300–1000 nm, which is an ideal nanofiber in the formation of hydrogel.

### 3.2. Mechanical Properties of the Hydrogels

**Figure 3:**
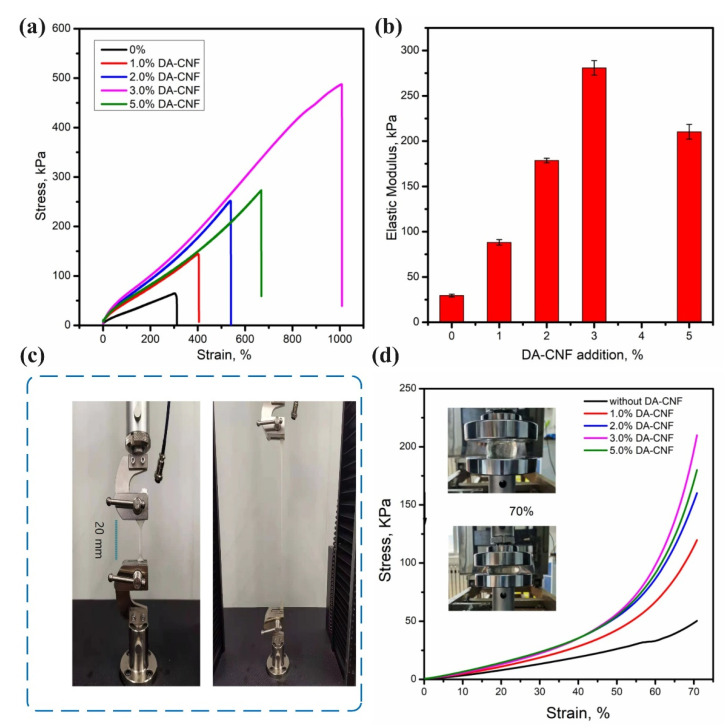
(**a**)Tensile curves of hydrogel with different DA-CNF content. (**b**) Elastic modulus and toughness calculated from the stress–strain curves. (**c**) Images of hydrogel showing excellent mechanical properties. (**d**) Compression properties of hydrogel.

To further evaluate the effect of DA-CNFs on the hydrogels, the mechanical properties of the P(AM-co-AMPS)/PA/DA-CNF hydrogel were determined (Figure 3). Figure 3a shows the uniaxial tensile stress–strain curves of different hydrogels. The addition of DA-CNFs significantly improved the fracture strength of the hydrogel compared to the hydrogels prepared with CNFs. Moreover, increasing the DA-CNF content, the fracture strength of the hydrogel was also increased. Similarly, the toughness and elastic modulus of the hydrogel prepared with DA-CNFs [P(AM-co-AMPS)/PA/DA-CNF] were also increased with increasing the DA-CNF loading. At a DA-CNF loading of 3 wt%, the fracture strength, toughness, and elastic modulus were 485 kPa, 210, and 280 kPa, respectively (Figure 3a–c). Moreover, it is noteworthy that the hydrogel prepared with 1 wt% of DA-CNFs also demonstrated much better mechanical performance than that of the hydrogel prepared without CNFs. These results are likely due to the chemical nature and interactions within the hydrogels. In addition, when the hydrogel is subjected to external forces, hydrogen bonds absorb and disperse stress as “sacrificial bonds” [25]. Compared to without DA-CNF, in addition to a large number of hydroxyl groups on the CNF surface itself, the nanofibers chain also contains carboxyl groups, which may lead to the formation of more hydrogen bonds between the fiber and the polymer chain. 

The compressive properties of P(AAm-co-AMPS)-PA, P(AAm-co-AMPS)/PA-DA-CNF hydrogels were also compared. As shown in Figure 3d, the hydrogel with more DA-CNF addition performed better than without DA-CNF addition, the compressive strength was increased with the DA-CNF addition increase. For instance, the compressive strength of hydrogel with 3% DA-CNF addition reached 229 kPa, which was 5-times that of the hydrogel without the DA-CNF addition. Compressive strength of the hydrogel without the DA-CNF addition was only 50 kPa. These observations are partly due to the fact that cellulose nanofibers act as supporting skeletons to prevent the hydrogel structure from collapsing rapidly [26,27]. Of course, the cross-linking Schiff bond structure between DA-CNF and the polyamine chain may also strengthen the internal network of the hydrogel.

**Figure 4:**
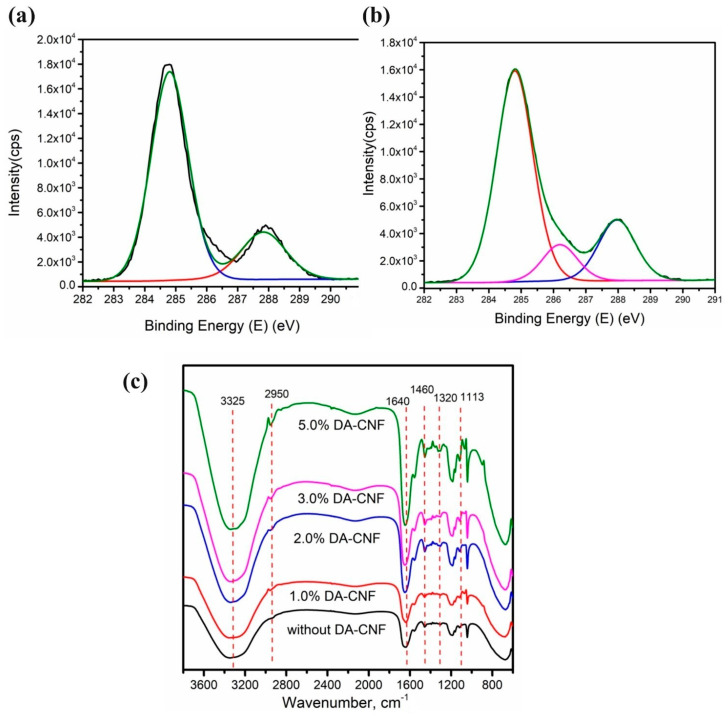
Characteristics of DA-CNF based hydrogel. (**a**) XPS of hydrogel without DA-CNF. (**b**) XPS of hydrogel with 3.0% DA-CNF. (**c**) FTIR spectra of hydrogel.

In order to prove the crosslinking situation of the double network hydrogels, the P(AM-co-AMPS)/PA/DA-CNF hydrogel was characterized by Fourier transform infrared (FTIR) spectra and XPS. Figure 4a, b shows, compared with the hydrogel without DA-CNF addition, when DA-CNF addition was 3%, a new signal at 286.3 eV was observed, which is the C=N contribution arising from DA-CNF and polyamine through the Schiff base reaction. It can prove that the aldehyde group on the surface of the DA-CNF reacted with the amine group of the polyamine polymer during the hydrogel formation, and formed a second network in the hydrogel. Figure 4c was Fourier transform infrared (FTIR) spectra of P(AM-co-AMPS)/PA/DA-CNF hydrogel. With the increase in DA-CNF addition, a new absorption peak of the hydrogel at 2950 cm^−1^, it is the absorption peak of CH_2_ or CH_3_ in polymer brushes on the surface of the CNFs. In addition, a new absorption peak at 1320 cm^−1^ is the -C=N absorption peak formed by the reaction between DA-CNF with polyamine, which proves that the aldehyde group on the surface of DA-CNF reacted with the amine group in the polyamine polymers.

### 3.3. Swelling Behavior and Texture Intensity in High Salinity Solutions

**Figure 5:**
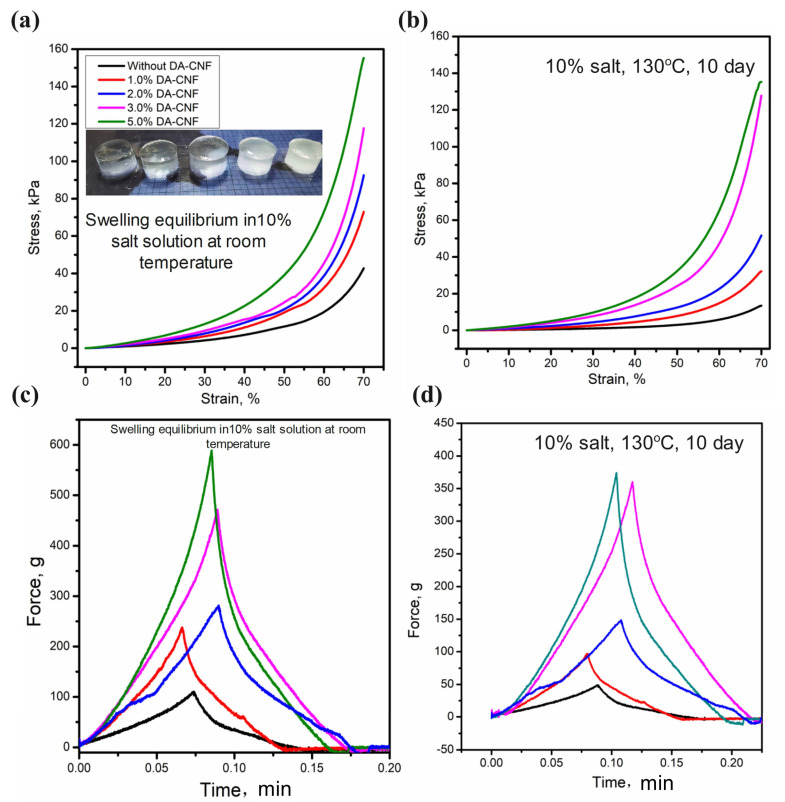
Compressive properties of swelling hydrogels. (**a**) Compressive properties of swelling hydrogels before aging; (**b**) Compressive properties of swelling hydrogels after aged 10 days; (**c**) Bloom strength of hydrogels before aging; (**d**) Bloom strength of hydrogels after aged 10 days.

To further evaluate the effect of DA-CNFs on the hydrogel mechanical properties in swelling in salt solution, the compressive properties of swelling P(AM-co-AMPS)/PA/DA-CNF hydrogel before and after aging in 130 °C temperature and 10% salt solution were determined. As shown in Figure 5a the P(AM-co-AMPS)/PA/DA-CNF (0 to 5 wt%), after the expansion equilibrium, adding DA-CNF can significantly improve the compression properties of the gel, when the gel is aged for 10 days at 10% salt solution and 130 °C, the compressive strength of hydrogel with 5% and 3% DA-CNF can still be close to the pre-aging. For example, before high temperature aging, the compressive strength of hydrogel with 0% DA-CNF was 42 kPa, but when the expansion hydrogel was aging at 130 °C, 10% salt solution for 10 days, the compressive strength of hydrogel was decreased to 10.5 kPa; for the hydrogel with 5% DA-CNF addition, the compressive strength of hydrogel matained at 140 Kpa after aging at 130 °C, 10% salt solution for 10 days. Temperature and salt resistance results shows that the addition of DA-CNF can improve the stability of hydrogel at high temperature and salt solution, due to the DA-CNF addition: (1) played a fundamental role and as the “support” of the polymer network; (2) the double network crosslinking. The mechanical strength of hydrogels would be affected after swelling, thus the texture tests were performed when the swelling ratios of all hydrogels almost equaled (around 20) in salt solution. Force curves from texture tests of hydrogels are shown in Figure 5c,d. In this work, the texture intensity of the hydrogel is evaluated by measuring the hardness of the hydrogel, the hydrogel hardness is defined as the peak of the force curve when the probe is pressed to 70% of the thickness of the hydrogel. Incorporation of DA-CNF (1 wt% to 5 wt%) apparently reinforced the hardness of hydrogels. In addition, the texture intensity of the hydrogel with complete swelling equilibrium was tested (Figure 5c,d), P(AM-co-AMPS)/PA/DA-CNF (0 to 5 wt%) still showed the best texture intensity. In general, hydrogels cross-linked by double network suffer severe mechanical damage in salt solutions, and this phenomenon is evident in the nanocomposite hydrogel [28]. In conclusion, P(AM-co-AMPS)/PA/DA-CNF has better effect than P(AM-co-AMPS) in texture intensity after swelling is more outstanding than P(AM-co-AMPS).

**Figure 6:**
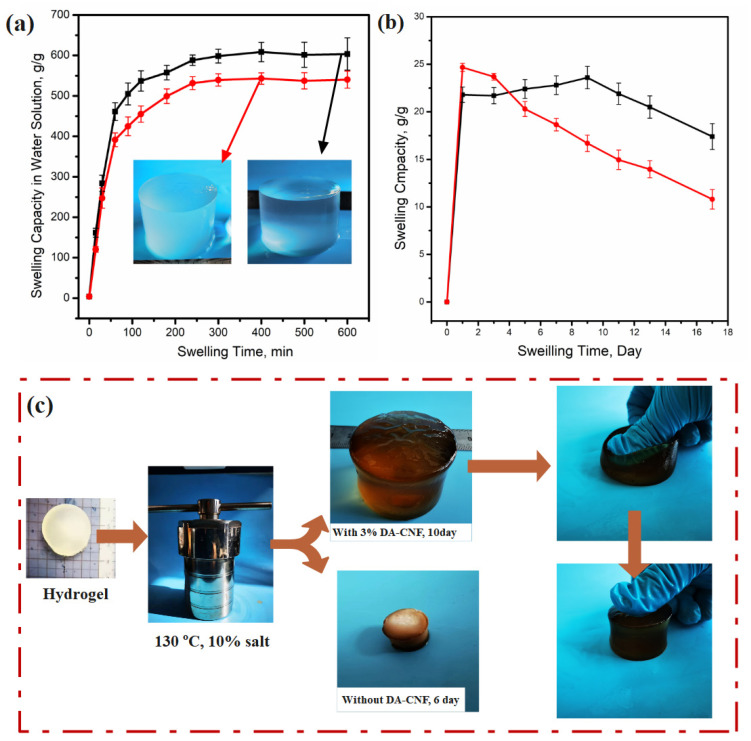
The swelling kinetics for all hydrogels in high-temperature and salt environment. (**a**) The equilibrium swelling ratio of all hydrogels in deionized water. (**b**) The equilibrium swelling ratio of all hydrogels in 10% salt solution. (**c**) Image shows the hydrogel aging in the salt solution.

To explore the feasibility of P(AM-co-AMPS)/PA/DA-CNF as a compressive material in a high-salinity environment (such as oil exploitation plugging agent), the swelling behavior of these hydrogels at high temperature was studied. The swelling kinetics for all hydrogels in high salinity solution (10 wt%) are depicted in Figure 6a. Obviously, all hydrogels exhibited a similar tendency of swelling kinetics—the swelling ratio increased remarkably during the initial 12 h and then tended to flat afterwards. Hydrogels containing DA-CNF had a lower equilibrium swelling ratio than that of the hydrogel without CNF in both deionized water and high salinity solution (Figure 6b), which can be attributed to the rigid network of CNF that limited the relaxation of the polymer chains [29]. The equilibrium swelling ratio of P(AM-co-AMPS)/PA/DA-CNF in salt solution was slightly higher than that of P(AM-Co-AMPS)/CNF in same CNF content. 

Recently, Ge [30,31,32] proposed a chemical and physical dual-crosslinking strategy to prepare a stretchable hydrogel composite that can be used in stretchable ionotronic devices, with ultrafast gelation and high adhesion in harsh environments. Gong [33,34,35] prepared a stretchable hydrogel composite that can be used in high-efficiency inverted polymer solar cells, and has an artificially intelligent optoelectronic skin with anisotropic electrical and optical responses and artificial bionic skin sensing. The above mentioned multifunctional hydrogels focus on the field of artificial skin sensing and stretchable electronic applications. Compared with the literature, the hydrogel prepared in this study is stable in high temperature and salt resistant, so it has potential for application as water plugging for the petroleum exploitation field. In order to study the potential application value of P(AM-co-AMPS)/PA/DA-CNF as a petroleum extraction plugging hydrogel, the stability of P(AM-co-AMPS)/PA/DA-CNF under high salinity (10%) and high temperature (130 °C) was tested. As shown in Figure 6, all hydrogels swelled quickly in salt solution within 10 h, compared with hydrogel without DA-CNF, P(AM-co-AMPS)/PA/DA-CNF swelled slowly. As shown in Figure 6c, for the P(AM-co-AMPS)/PA/DA-CNF with 3% DA-CNF, after 16 days aging, the weight of the hydrogel was decreased to 75.1%. These results demonstrated that the tight cross-linking between DACNF and polyamine chains improves the stability of hydrogels in high-salinity and high-temperature environments. With the double network of the P(AM-co-AMPS)/PA/DA-CNF has excellent decomposition stability and strength in the high-temperature and high-salinity environment, which gives it the potential for practical application in the petroleum exploitation field.

## 4. Conclusions

In this study, DA-CNF, polyamine, AM, and AMPS aqueous solution were mixed together to prepare the double network hydrogel in one step. The dialdehyde groups on the surface of DA-CNF and amino group of polyamine could form Schiff base for a network and another network was crosslinked by AM and AMPS. The tensile fracture strength, elastic modulus, toughness, and dissipative energy of P(AM-co-AMPS)/PA/DA-CNF showed the good performance under the synergistic effect of hydrogen bond, physical entanglement and ion crosslinking between DA-CNF and chains. The reversible Schiff base bonds between DA-CNF and polyamine give hydrogels a certain self-recovery ability. What is more noteworthy is that the traditional gel system has poor salt resistance in oil exploitation plugging, while the hydrogel prepared in this paper still shows the best structural strength under high salinity conditions, overcoming the shortcomings of traditional gel, which provides a theoretical basis for the application of hydrogels in oil exploitation, which gives them potential applications in high salinity fields such as oil exploitation plugging.

## Data Availability

The data measured for this study are available on request from the corresponding author.

## References

[1] Bashari A., Shirvan A.R., Shakeri M. (2018). Cellulose-based hydrogels for personal care products. Polym. Adv. Technol..

[2] Liu C., Lei F., Li P., Jiang J., Wang K. (2020). Borax crosslinked fenugreek galactomannan hydrogel as potential water-retaining agent in agriculture. Carbohydr. Polym..

[3] Gao J., Yuan Y., Yu Q., Yan B., Qian Y., Wen J., Ma C., Jiang S., Wang X., Wang N. (2020). Bio-inspired antibacterial cellulose paper–poly (amidoxime) composite hydrogel for highly efficient uranium (vi) capture from seawater. Chem. Commun..

[4] Tavakoli J., Tang Y. (2017). Hydrogel based sensors for biomedical applications: An updated review. Polymers.

[5] Zareie C., Bahramian A.R., Sefti M.V., Salehi M.B. (2019). Network-gel strength relationship and performance improvement of polyacrylamide hydrogel using nano-silica; with regards to application in oil wells conditions. J. Mol. Liq..

[6] Peak C.W., Wilker J.J., Schmidt G. (2013). A review on tough and sticky hydrogels. Colloid Polym. Sci..

[7] Wang L., Long Y., Ding H., Geng J., Bai B. (2017). Mechanically robust re-crosslinkable polymeric hydrogels for water management of void space conduits containing reservoirs. Chem. Eng. J..

[8] Pu J., Zhou J., Chen Y., Bai B. (2017). Development of thermos-transformable controlled hydrogel for enhancing oil recovery. Energy Fuels.

[9] Gu Z., Huang K., Luo Y., Zhang L., Kuang T., Chen Z., Liao G. (2018). Double network hydrogel for tissue engineering. Wiley Interdiscip. Rev. Nanomed. Nanobiotechnol..

[10] Schexnailder P., Schmidt G. (2009). Nanocomposite polymer hydrogels. Colloid Polym. Sci..

[11] Satarkar N.S., Biswal D., Hilt J.Z. (2010). Hydrogel nanocomposites: A review of applications as remote controlled biomaterials. Soft Matter.

[12] Haraguchi K. (2007). Nanocomposite hydrogels. Curr. Opin. Solid State Mater. Sci..

[13] Haraguchi K., Takehisa T. (2002). Nanocomposite hydrogels: A unique organic–inorganic network structure with extraordinary mechanical, optical, and swelling/de-swelling properties. Adv. Mater..

[14] Kim J.H., Shim B.S., Kim H.S., Lee Y.J., Min S.K., Jang D., Abas Z., Kim J. (2015). Review of nanocellulose for sustainable future materials. Int. J. Precis. Eng. Manuf. Green Technol..

[15] Luo H., Cha R., Li J., Hao W., Zhang Y., Zhou F. (2019). Advances in tissue engineering of nanocellulose-based scaffolds: A review. Carbohydr. Polym..

[16] De France K.J., Hoare T., Cranston E.D. (2017). Review of hydrogels and aerogels containing nanocellulose. Chem. Mater..

[17] Nascimento D.M., Nunes Y.L., Figueirêdo M.C., de Azeredo H.M., Aouada F.A., Feitosa J.P., Dufresne A., Rosa M.F. (2018). Nanocellulose nanocomposite hydrogels: Technological and environmental issues. Green Chem..

[18] Huang S., Zhao Z., Feng C., Mayes E., Yang J. (2018). Nanocellulose reinforced P (AAm-co-AAc) hydrogels with improved mechanical properties and biocompatibility. Compos. Part A Appl. Sci. Manuf..

[19] Liu Q., Liu J., Qin S., Pei Y., Zheng X., Tang K. (2020). High mechanical strength gelatin composite hydrogels reinforced by cellulose nanofibrils with unique beads-on-a-string morphology. Int. J. Biol. Macromol..

[20] Madivoli E.S., Kareru P.G., Gachanja A.N., Mugo S.M., Makhanu D.S. (2019). Synthesis and characterization of dialdehyde cellulose nanofibers from *O. sativa* husks. SN Appl. Sci..

[21] Wang Y., Xiao G., Peng Y., Chen L., Fu S. (2019). Effects of cellulose nanofibrils on dialdehyde carboxymethyl cellulose based dual responsive self-healing hydrogel. Cellulose.

[22] Sun B., Hou Q., Liu Z., Ni Y. (2015). Sodium periodate oxidation of cellulose nanocrystal and its application as a paper wet strength additive. Cellulose.

[23] Ye Y., Zhang Y., Chen Y., Han X., Jiang F. (2020). Cellulose nanofibrils enhanced, strong, stretchable, freezing-tolerant ionic conductive organohydrogel for multi-functional sensors. Adv. Funct. Mater..

[24] Chen W., Yu H., Liu Y., Chen P., Zhang M., Hai Y. (2011). Individualization of cellulose nanofibers from wood using high-intensity ultrasonication combined with chemical pretreatments. Carbohydr. Polym..

[25] Zhang J., Liu T., Liu Z., Wang Q. (2019). Facile fabrication of tough photocrosslinked polyvinyl alcohol hydrogels with cellulose nanofibrils reinforcement. Polymer.

[26] Liu S., Oderinde O., Hussain I., Yao F., Fu G. (2018). Dual ionic cross-linked double network hydrogel with self-healing, conductive, and force sensitive properties. Polymer.

[27] Hu J., Wu Y., Yang Q., Zhou Q., Hui L., Liu Z., Ding D. (2022). One-pot freezing-thawing preparation of cellulose nanofibrils reinforced polyvinyl alcohol based ionic hydrogel strain sensor for human motion monitoring. Carbohydr. Polym..

[28] Gao H., Mao J., Cai Y., Li S., Fu Y., Liu X., Liang H., Zhao T., Liu M., Jiang L. (2021). Euryhaline hydrogel with constant swelling and salinity-enhanced mechanical Strength in a Wide Salinity Range. Adv. Funct. Mater..

[29] Mahfoudhi N., Boufi S. (2016). Poly (acrylic acid-co-acrylamide)/cellulose nanofibrils nanocomposite hydrogels: Effects of CNFs content on the hydrogel properties. Cellulose.

[30] Ge G., Mandal K., Haghniaz R., Li M., Xiao X., Carlson L., Jucaud V., Dokmeci M.R., Ho G.W., Khademhosseini A. (2023). Deep Eutectic Solvents-Based Ionogels with Ultrafast Gelation and High Adhesion in Harsh Environments. Adv. Funct. Mater..

[31] Ge G., Wang Q., Zhang Y.Z., Alshareef H.N., Dong X. (2021). 3D printing of hydrogels for stretchable ionotronic devices. Adv. Funct. Mater..

[32] Ge G., Zhang Y.Z., Zhang W., Yuan W., El-Demellawi J.K., Zhang P., Alshareef H.N. (2021). Ti_3_C_2_T_x_MXene-Activated Fast Gelation of Stretchable and Self-Healing Hydrogels: A Molecular Approach. ACS Nano.

[33] Gong Y., Zhang Y.Z., Fang S., Sun Y., Niu J., Lai W.Y. (2022). Wireless Human–Machine Interface Based on Artificial Bionic Skin with Damage Reconfiguration and Multisensing Capabilities. ACS Appl. Mater. Interfaces.

[34] Gong Y., Zhang Y.Z., Fang S., Liu C., Niu J., Li G., Lai W.Y. (2022). Artificial intelligent optoelectronic skin with anisotropic electrical and optical responses for multi-dimensional sensing. Appl. Phys. Rev..

[35] Gong Y., Zhang J., Du B., Wang M., Lai W.Y., Huang W. (2019). Design, synthesis, and postvapor treatment of neutral fulleropyrrolidine electron-collecting interlayers for high-efficiency inverted polymer solar cells. ACS Appl. Electron. Mater..

